# Supplementary material for: Dental characters used in phylogenetic analyses of mammals show higher rates of evolution, but not reduced independence
Source: PeerJ. 2020 Mar 24;8:e8744. doi: 10.7717/peerj.8744 (PMC7100591; doi:10.7717/peerj.8744)
Supplement: Supplemental Information 1 [file peerj-08-8744-s001.docx]

Table S1. Results of GLS analyses of Pagel’s λ (phylogenetic signal of character partitions) in cetartiodactyls. Rows coloured are those where the partition best fits the H1 model (partition has a different lambda value to all others); blue= lower, red=higher.

| Partition | Median λ | GLS Coefficient | lnL (null) | lnL (H1) | AIC (null) | AIC (H1) | Akaike weights (null) | Akaike weights (H1) |
| --- | --- | --- | --- | --- | --- | --- | --- | --- |
| Teeth | 0.82 | -0.15 | -197.68 | -196.3 | 399.35 | 398.58 | 0.41 | 0.59 |
| Skull | 1 | 0.05 | -197.68 | -199.2 | 399.35 | 404.43 | 0.93 | 0.07 |
| Vertebrae | 1 | 0.06 | -197.68 | -198.7 | 399.35 | 403.45 | 0.89 | 0.11 |
| Forelimb | 0.91 | 0.12 | -197.68 | -198.1 | 399.35 | 402.39 | 0.82 | 0.18 |
| Hindlimb | 0.89 | -0.12 | -197.68 | -198.3 | 399.35 | 402.68 | 0.84 | 0.16 |
| Soft tissue | 1 | 0.12 | -197.68 | -198.4 | 399.35 | 402.80 | 0.85 | 0.15 |

Table S2. Results of GLS analyses of Akaike weight support for the equal rates (ER) model of character evolution in artiodactyls. No partitions fit the H1 model (partition has a different rate value to all others) better than the null

| Partition | Median weight | GLS Coefficient | lnL (null) | lnL (H1) | AIC (null) | AIC (H1) | Akaike weights (null) | Akaike weights (H1) |
| --- | --- | --- | --- | --- | --- | --- | --- | --- |
| Teeth | 0.71 | -0.01 | 102.3 | 99.57 | -200.6 | -193.1 | 0.98 | 0.02 |
| Skull | 0.70 | -0.03 | 102.3 | 100.21 | -200.6 | -194.4 | 0.96 | 0.04 |
| Vertebrae | 0.76 | 0.05 | 102.3 | 100.75 | -200.6 | -195.5 | 0.93 | 0.07 |
| Forelimb | 0.71 | 0.03 | 102.3 | 100.35 | -200.6 | -194.7 | 0.95 | 0.05 |
| Hindlimb | 0.75 | 0.04 | 102.3 | 100.54 | -200.6 | -195.1 | 0.94 | 0.06 |
| Soft tissue | 0.73 | 0.03 | 102.3 | 100.34 | -200.6 | -194.7 | 0.95 | 0.05 |

Table S3. Results of GLS analyses of Akaike weight support for the independent model of character evolution in artiodactyls. Rows coloured are those where the partition best fits the H1 model (partition has a different rate value to all others); blue= lower, red=higher.

| Partition | Median weight | GLS Coefficient | lnL (null) | lnL (H1) | AIC (null) | AIC (H1) | Akaike weights (null) | Akaike weights (H1) |
| --- | --- | --- | --- | --- | --- | --- | --- | --- |
| Teeth | 0.80 | -0.04 | 1258.4 | 1256.7 | -2513 | -2507 | 0.94 | 0.06 |
| Skull | 0.70 | 0.12 | 1258.4 | 1313.2 | -2513 | -2620 | ~0 | ~1 |
| Vertebrae | 0.66 | -0.06 | 1258.4 | 1257.1 | -2513 | -2508 | 0.91 | 0.09 |
| Forelimb | 0.57 | -0.16 | 1258.4 | 1327.3 | -2513 | -2649 | ~0 | ~0.1 |
| Hindlimb | 0.81 | 0.18 | 1258.4 | 1258.1 | -2513 | -2510 | 0.78 | 0.22 |
| Soft tissue | 0.64 | -0.02 | 1258.4 | 1256.0 | -2513 | -2506 | 0.97 | 0.03 |

Table S4. Results of GLS analyses of rates of character evolution in artiodactyls. Rows coloured are those where the partition best fits the H1 model (partition has a different rate value to all others); blue= lower, red=higher.

| Partition | Median rate | GLS Coefficient | lnL (null) | lnL (H1) | AIC (null) | AIC (H1) | Akaike weights (null) | Akaike weights (H1) |
| --- | --- | --- | --- | --- | --- | --- | --- | --- |
| Teeth | 0.0043 | 0.27 | -385.41 | -383.2 | 779.29 | 774.83 | 0.23 | 0.77 |
| Skull | 0.0044 | 0.03 | -385.41 | -386.9 | 779.29 | 774.83 | 0.92 | 0.08 |
| Vertebrae | 0.0027 | -0.33 | -385.41 | -384.8 | 779.29 | 774.83 | 0.60 | 0.40 |
| Forelimb | 0.0029 | -0.17 | -385.41 | -385.8 | 779.29 | 774.83 | 0.80 | 0.20 |
| Hindlimb | 0.0042 | -0.001 | -385.41 | -386.2 | 779.29 | 774.83 | 0.85 | 0.15 |
| Soft tissue | 0.0017 | -0.55 | -385.41 | -382.1 | 779.29 | 774.83 | 0.09 | 0.91 |

Table S5. Results of GLS analyses of Pagel’s λ (phylogenetic signal of character partitions) in carnivorans. Rows coloured are those where the partition best fits the H1 model (partition has a different lambda value to all others); blue= lower,

| Partition | Median λ | GLS Coefficient | lnL (null) | lnL (H1) | AIC (null) | AIC (H1) | Akaike weights (null) | Akaike weights (H1) |
| --- | --- | --- | --- | --- | --- | --- | --- | --- |
| Teeth | ~0 | -0.38 | -64.55 | -59.97 | 133.10 | 125.94 | 0.03 | 0.97 |
| Skull | 1 | 0.30 | -64.55 | -61.86 | 133.10 | 129.72 | 0.16 | 0.84 |
| Vertebrae | 1 | 0.48 | -64.55 | -63.86 | 133.10 | 133.72 | 0.58 | 0.42 |
| Forelimb | ~0 | -0.52 | -64.55 | -63.76 | 133.10 | 133.53 | 0.55 | 0.45 |
| Hindlimb | 0.5 | -0.02 | -64.55 | -64.66 | 133.10 | 135.31 | 0.75 | 0.25 |
| Soft tissue | 1 | 0.23 | -64.55 | -64.56 | 133.10 | 135.13 | 0.73 | 0.27 |

Table S6. Results of GLS analyses of Akaike weight support for the equal rates (ER) model of character evolution in carnivorans. No partitions fit the H1 model (partition has a different rate value to all others) better than the null

| Partition | Median weight | GLS Coefficient | lnL (null) | lnL (H1) | AIC (null) | AIC (H1) | Akaike weights (null) | Akaike weights (H1) |
| --- | --- | --- | --- | --- | --- | --- | --- | --- |
| Teeth | ~1 | 0.07 | -24.72 | -25.99 | 53.45 | -57.98 | 0.91 | 0.09 |
| Skull | 0.79 | -0.05 | -24.72 | -26.17 | 53.45 | -58.34 | 0.92 | 0.08 |
| Vertebrae | 0.74 | 0.05 | -24.72 | -24.95 | 53.45 | -55.91 | 0.77 | 0.23 |
| Forelimb | 0.71 | -0.03 | -24.72 | -24.95 | 53.45 | -55.90 | 0.77 | 0.23 |
| Hindlimb | 0.75 | 0.01 | -24.72 | -25.29 | 53.45 | -56.59 | 0.83 | 0.17 |
| Soft tissue | 0.69 | -0.03 | -24.72 | -25.61 | 53.45 | -57.22 | 0.87 | 0.13 |

Table S7. Results of GLS analyses of Akaike weight support for the independent model of character evolution in carnivorans. No partitions fit the H1 model (partition has a different rate value to all others) better than the null

| Partition | Median weight | GLS Coefficient | lnL (null) | lnL (H1) | AIC (null) | AIC (H1) | Akaike weights (null) | Akaike weights (H1) |
| --- | --- | --- | --- | --- | --- | --- | --- | --- |
| Teeth | 0.83 | 0.042 | 153.76 | 152.02 | -303.5 | -298.0 | 0.94 | 0.06 |
| Skull | 0.79 | -0.039 | 153.76 | 151.97 | -303.5 | -297.9 | 0.94 | 0.06 |
| Vertebrae | NA | NA | NA | NA | NA | NA | NA | NA |
| Forelimb | NA | NA | NA | NA | NA | NA | NA | NA |
| Hindlimb | 0.74 | 0.023 | 153.76 | 152.91 | -303.5 | -299.8 | 0.86 | 0.14 |
| Soft tissue | 0.71 | 0.028 | 153.76 | 152.09 | -303.5 | -298.2 | 0.93 | 0.06 |

Table S8. Results of GLS analyses of rates of character evolution in carnivorans. No partitions fit the H1 model (partition has a different rate value to all others) better than the null.

| Partition | Median rate | GLS Coefficient | lnL (null) | lnL (H1) | AIC (null) | AIC (H1) | Akaike weights (null) | Akaike weights (H1) |
| --- | --- | --- | --- | --- | --- | --- | --- | --- |
| Teeth | 0.0051 | 0.014 | -62.86 | -64.10 | 129.71 | 134.21 | 0.90 | 0.10 |
| Skull | 0.0046 | 0.002 | -62.86 | -64.17 | 129.71 | 134.34 | 0.91 | 0.09 |
| Vertebrae | 0.0045 | -0.140 | -62.86 | -62.60 | 129.71 | 131.21 | 0.68 | 0.32 |
| Forelimb | 0.0080 | 0.110 | -62.86 | -62.62 | 129.71 | 131.24 | 0.68 | 0.32 |
| Hindlimb | 0.0022 | -0.469 | -62.86 | -62.09 | 129.71 | 130.18 | 0.56 | 0.44 |
| Soft tissue | 0.0032 | 0.17 | -62.86 | -63.09 | 129.71 | 132.17 | 0.77 | 0.23 |

Table S9. Results of GLS analyses of Pagel’s λ (phylogenetic signal of character partitions) in Primates. Rows coloured are those where the partition best fits the H1 model (partition has a different lambda value to all others); blue= lower, red=higher.

| Partition | Median λ | GLS Coefficient | lnL (null) | lnL (H1) | AIC (null) | AIC (H1) | Akaike weights (null) | Akaike weights (H1) |
| --- | --- | --- | --- | --- | --- | --- | --- | --- |
| Teeth | 1 | 0.11 | -6.44 | -639.6 | 1292.6 | 1285.2 | 0.02 | 0.98 |
| Skull | 1 | 0.01 | -6.44 | -646.7 | 1292.6 | 1299.3 | 0.97 | 0.03 |
| Vertebrae | 0.91 | -0.13 | -6.44 | -644.7 | 1292.6 | 1295.5 | 0.80 | 0.20 |
| Forelimb | 0.96 | -0.06 | -6.44 | -645.6 | 1292.6 | 1297.3 | 0.91 | 0.09 |
| Hindlimb | 1 | 0.07 | -6.44 | -644.5 | 1292.6 | 1295.1 | 0.77 | 0.23 |
| Soft tissue | 0.91 | -0.20 | -6.44 | -630.6 | 1292.6 | 1267.2 | ~0 | 1 |

Table S10. Results of GLS analyses of Akaike weight support for the equal rates (ER) model of character evolution in primates. Rows coloured are those where the partition best fits the H1 model (partition has a different rate value to all others); blue= lower, red=higher.

| Partition | Median weight | GLS Coefficient | lnL (null) | lnL (H1) | AIC (null) | AIC (H1) | Akaike weights (null) | Akaike weights (H1) |
| --- | --- | --- | --- | --- | --- | --- | --- | --- |
| Teeth | 1 | 0.05 | 319.7 | 326.8 | -635.4 | -647.6 | ~0 | ~1 |
| Skull | 0.80 | -0.03 | 319.7 | 318.0 | -635.4 | -629.9 | 0.94 | 0.06 |
| Vertebrae | 0.79 | -0.01 | 319.7 | 317.4 | -635.4 | -628.8 | 0.96 | 0.04 |
| Forelimb | 0.79 | -0.07 | 319.7 | 322.2 | -635.4 | -638.4 | 0.18 | 0.82 |
| Hindlimb | 0.78 | -0.04 | 319.7 | 321.5 | -635.4 | -637.1 | 0.30 | 0.70 |
| Soft tissue | 0.80 | 0.02 | 319.7 | 317.2 | -635.4 | -628.4 | 0.97 | 0.03 |

Table S11. Results of GLS analyses of Akaike weight support for the independent model of character evolution in primates. Rows coloured are those where the partition best fits the H1 model (partition has a different rate value to all others); blue= lower, red=higher.

| Partition | Median weight | GLS Coefficient | lnL (null) | lnL (H1) | AIC (null) | AIC (H1) | Akaike weights (null) | Akaike weights (H1) |
| --- | --- | --- | --- | --- | --- | --- | --- | --- |
| Teeth | 0.80 | -0.045 | 9332.2 | 9586 | -18660 | -19166 | ~0 | ~1 |
| Skull | 0.78 | -0.029 | 9332.2 | 9376 | -18660 | -18746 | ~0 | ~1 |
| Vertebrae | 0.78 | -0.043 | 9332.2 | 9332 | -18660 | 18657 | 0.83 | 0.17 |
| Forelimb | 0.78 | -0.017 | 9332.2 | 9335 | -18660 | -18664 | 0.13 | 0.87 |
| Hindlimb | 0.78 | -0.044 | 9332.2 | 9457 | -18660 | -18908 | ~0 | ~1 |
| Soft tissue | 0.88 | 0.130 | 9332.2 | 10772 | -18660 | -21540 | ~0 | ~1 |

Table S12. Results of GLS analyses of rates of character evolution in primates. Rows coloured are those where the partition best fits the H1 model (partition has a different rate value to all others); blue= lower, red=higher.

| Partition | Median rate | GLS Coefficient | lnL (null) | lnL (H1) | AIC (null) | AIC (H1) | Akaike weights (null) | Akaike weights (H1) |
| --- | --- | --- | --- | --- | --- | --- | --- | --- |
| Teeth | 0.0031 | -0.64 | -1385 | -1316 | 2774.0 | 2638.0 | ~0 | ~1 |
| Skull | 0.0040 | -0.34 | -1385 | -1376 | 2774.0 | 2757.3 | ~0 | ~1 |
| Vertebrae | 0.0052 | -0.01 | -1385 | -1386 | 2774.0 | 2777.2 | 0.83 | 0.17 |
| Forelimb | 0.0054 | 0.00 | -1385 | -1386 | 2774.0 | 2778.8 | 0.92 | 0.08 |
| Hindlimb | 0.0046 | -0.24 | -1385 | -1382 | 2774.0 | 2769.3 | 0.09 | 0.91 |
| Soft tissue | 0.0072 | 1.56 | -1385 | -1059 | 2774.0 | 2124.6 | ~0 | ~1 |

Table S13. Results of GLS analyses of Pagel’s λ (phylogenetic signal of character partitions) in marsupials. No partitions fit the H1 model (partition has a different rate value to all others) better than the null

| Partition | Median λ | GLS Coefficient | lnL (null) | lnL (H1) | AIC (null) | AIC (H1) | Akaike weights (null) | Akaike weights (H1) |
| --- | --- | --- | --- | --- | --- | --- | --- | --- |
| Teeth | 1 | 0.113 | -84.84 | -85.40 | 173.67 | 176.79 | 0.83 | 0.17 |
| Skull | 1 | 0.043 | -84.84 | -86.51 | 173.67 | 179.03 | 0.94 | 0.06 |
| Vertebrae | 0.96 | -0.082 | -84.84 | -85.91 | 173.67 | 177.82 | 0.89 | 0.11 |
| Forelimb | 1 | -0.085 | -84.84 | -85.81 | 173.67 | 177.63 | 0.88 | 0.12 |
| Hindlimb | 1 | 0.049 | -84.84 | -86.44 | 173.67 | 178.87 | 0.93 | 0.07 |
| Soft tissue | 0.93 | -0.112 | -84.84 | -85.43 | 173.67 | 176.85 | 0.83 | 0.17 |

Table S14. Results of GLS analyses of Akaike weight support for the equal rates (ER) model of character evolution in marsupials. Rows coloured are those where the partition best fits the H1 model (partition has a different rate value to all others), red=higher.

| Partition | Median weight | GLS Coefficient | lnL (null) | lnL (H1) | AIC (null) | AIC (H1) | Akaike weights (null) | Akaike weights (H1) |
| --- | --- | --- | --- | --- | --- | --- | --- | --- |
| Teeth | 0.75 | 0.032 | 25.68 | 23.77 | -47.37 | -41.54 | 0.95 | 0.05 |
| Skull | 0.71 | -0.002 | 25.68 | 23.21 | -47.37 | -40.42 | 0.97 | 0.03 |
| Vertebrae | 0.75 | -0.019 | 25.68 | 23.58 | -47.37 | -41.15 | 0.96 | 0.04 |
| Forelimb | 0.98 | 0.20 | 25.68 | 26.90 | -47.37 | -47.79 | 0.45 | 0.55 |
| Hindlimb | 0.77 | 0.11 | 25.68 | 24.89 | -47.37 | -43.78 | 0.86 | 0.04 |
| Soft tissue | 0.69 | -0.039 | 25.68 | 23.88 | -47.37 | -41.76 | 0.95 | 0.06 |

Table S15. Results of GLS analyses of Akaike weight support for the independent model of character evolution in marsupials. Rows coloured are those where the partition best fits the H1 model (partition has a different rate value to all others); blue= lower.

| Partition | Median weight | GLS Coefficient | lnL (null) | lnL (H1) | AIC (null) | AIC (H1) | Akaike weights (null) | Akaike weights (H1) |
| --- | --- | --- | --- | --- | --- | --- | --- | --- |
| Teeth | 0.70 | -0.068 | 156.41 | 158.45 | -308.8 | -310.9 | 0.26 | 0.74 |
| Skull | 0.74 | 0.005 | 156.41 | 153.00 | -308.8 | -300.0 | 0.99 | 0.01 |
| Vertebrae | 0.74 | -0.0003 | 156.41 | 153.29 | -308.8 | -300.6 | 0.98 | 0.02 |
| Forelimb | 0.77 | 0.013 | 156.41 | 153.90 | -308.8 | -301.8 | 0.97 | 0.03 |
| Hindlimb | 0.78 | 0.034 | 156.41 | 155.02 | -308.8 | -304.0 | 0.92 | 0.08 |
| Soft tissue | NA | NA | NA | NA | NA | NA | NA | NA |

Table S16. Results of GLS analyses of rates of character evolution in marsupials. Rows coloured are those where the partition best fits the H1 model (partition has a different rate value to all others); blue= lower, red=higher.

| Partition | Median rate | GLS Coefficient | lnL (null) | lnL (H1) | AIC (null) | AIC (H1) | Akaike weights (null) | Akaike weights (H1) |
| --- | --- | --- | --- | --- | --- | --- | --- | --- |
| Teeth | 0.003 | -0.205 | -316.4 | -316.6 | 636.90 | 639.22 | 0.76 | 0.24 |
| Skull | 0.004 | 0.343 | -316.4 | -314.0 | 636.90 | 634.03 | 0.19 | 0.81 |
| Vertebrae | 0.004 | -0.015 | -316.4 | -317.3 | 636.90 | 640.60 | 0.86 | 0.14 |
| Forelimb | 0.003 | -0.095 | -316.4 | -317.1 | 636.90 | 640.26 | 0.84 | 0.16 |
| Hindlimb | 0.002 | -0.334 | -316.4 | -314.2 | 636.90 | 632.25 | 0.23 | 0.77 |
| Soft tissue | 0.006 | 0.822 | -316.4 | -313.1 | 636.90 | 639.88 | 0.09 | 0.91 |
